# Hemodialysis Modality and Mortality Outcomes among Incident Dialysis Patients: An International Cohort Study Comparing High-Volume Hemodiafiltration and Hemodialysis

**DOI:** 10.2215/CJN.0000001063

**Published:** 2026-05-14

**Authors:** Yan Zhang, Anke Winter, Linda H. Ficociello, Smriti Arya, Stefano Stuard, Len A. Usvyat, Kamyar Kalantar-Zadeh

**Affiliations:** 1 Renal Research Institute, New York, New York; 2 Fresenius Medical Care, Global Medical Office, Hong Kong, China; 3 Fresenius Medical Care Deutschland GmbH, Bad Homburg, Germany; 4 Fresenius Medical Care, Global Medical Office, Palazzo Pignano, Italy; 5 Harbor-UCLA Medical Center and The Lundquist Institute, Torrance, California

**Keywords:** dialysis, mortality

## Abstract

**Key Points:**

- High-volume hemodiafiltration was associated with a 20% lower all-cause mortality risk compared with hemodialysis in incident patients.
- High-volume hemodiafiltration was associated with a 29% lower cardiovascular mortality risk compared with hemodialysis in incident patients.
- Associations between high-volume hemodiafiltration and lower mortality were consistent across demographic and clinical subgroups.

**Background:**

Evidence for a survival benefit of hemodiafiltration (HDF) over high-flux hemodialysis largely comes from studies based on prevalent ESKD patients with longer dialysis exposure. By contrast, the effect of HDF on mortality of incident patients—those newly starting dialysis—remains less well understood.

**Methods:**

We analyzed data from 18,515 incident patients (dialysis vintage <3 months) treated between 2019 and 2022 at Fresenius Medical Care NephroCare Clinics. Patients were classified as HDF or hemodialysis on the basis of their predominant dialysis modality during the first year of follow-up (≥75% of sessions). To assess the effect of HDF in the early phase after treatment initiation, follow-up was limited to 2 years. Cox proportional hazards models with inverse probability of treatment weighting were applied to estimate all-cause and cardiovascular disease mortality risk.

**Results:**

Baseline characteristics between HDF and hemodialysis groups were comparable after inverse probability of treatment weighting. Over a median follow-up of 15.7 months (interquartile range, 6.4–24.0 months), HDF was associated with a lower risk of all-cause mortality compared with hemodialysis (11.7 versus 15.6 per 100 person-years; hazard ratio, 0.80; 95% confidence interval, 0.75 to 0.86). Furthermore, HDF was associated with a lower risk of cardiovascular disease mortality compared with hemodialysis (4.1 versus 6.7 per 100 person-years; hazard ratio, 0.71; 95% confidence interval, 0.63 to 0.80).

**Conclusions:**

In the large real-world cohort of incident patients with ESKD who are in the early phase of dialysis treatment, online HDF was associated with a significant survival advantage compared with conventional hemodialysis. These findings reinforce the potential clinical benefits of HDF and support early adoption of HDF upon dialysis initiation.

## Visual Abstract

**Figure:**
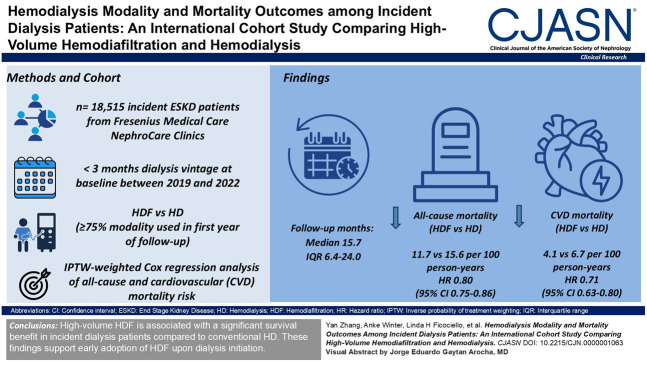

## Introduction

ESKD imposes a significant global health burden, with hemodialysis serving as the predominant life-sustaining KRT for millions worldwide.^1^ Despite technological advances, conventional hemodialysis remains limited in its capacity to effectively clear middle- and large–molecular-weight uremic toxins, contributing to persistent inflammation, accelerated cardiovascular disease (CVD), and high mortality rates.^2^ Hemodiafiltration (HDF) emerged as a promising alternative by combining diffusive and convective transport. It has demonstrated significant clinical benefits over conventional hemodialysis, predominantly in reducing mortality and cardiovascular events.^3,4^ The recent CONVective dialysis versus hemodialysis IN reducing mortality and Cardiovascular Events (CONVINCE) trial reported a 23% lower risk of death with high-volume HDF in a broad patient population treated in routine clinical practice.^5^

Recent meta-analyses focusing specifically on high-volume HDF suggest a potential mortality advantage compared with conventional high-flux hemodialysis (HF-HD).^3^ However, critical knowledge gaps persist as most randomized controlled trials (RCTs) and observational studies on HDF excluded incident patients,^3,6–8^ a known high-risk patient group.^9,10^

Incident patients on dialysis face a markedly elevated mortality and hospitalization risk, especially in the early stages after treatment initiation.^9,10^ These higher risks might be associated with comorbidities, older age, clinical symptoms, and suboptimal dialysis initiation, among others.^11^ Hence, the early phase after initiating dialysis is a critical period requiring close monitoring. Data specifically examining the effect of high-volume hemodiafiltration (HV-HDF) versus HF-HD on survival in this uniquely vulnerable population are scarce.

Furthermore, RCTs, although methodologically robust, may not fully reflect the effectiveness of HV-HDF when applied in routine clinical practice or real-world settings because of strict inclusion/exclusion criteria or protocol-driven care and potentially achieved lower convection volumes. Therefore, evaluating the comparative effectiveness of HV-HDF using robust real-world data in incident patients on dialysis is crucial. This study aims to address this gap and investigate whether the initiation of HV-HDF, compared with conventional hemodialysis, is associated with reduced mortality outcomes among incident patients on dialysis treated under routine care conditions.

## Methods

### Study Design and Population

This retrospective cohort study was conducted among in-center patients on dialysis treated in Fresenius Medical Care Europe, Middle East, and Africa NephroCare centers from January 1, 2019, to December 31, 2022. All patients' data were extracted from the European Clinical Database (EuCliD), a standardized electronic medical record system collecting real-world medical data of patients on dialysis in NephroCare clinics.^12,13^ Adult patients (≥18 years) who had newly initiated dialysis (dialysis vintage less than 3 months) at admission in NephroCare clinics were eligible for inclusion if they provided informed consent for the use of their pseudoanonymized data in secondary analyses. To evaluate the effect of HDF versus hemodialysis on mortality among incident patients with sufficient follow-up to establish dialysis modality and dialysis adequacy, a 30-day baseline period beginning from the time of initiation of treatment within NephroCare was defined and several prespecified exclusion criteria were applied. Patients were excluded if they met any of the following criteria: (*1*) being censored within the baseline period, including death, kidney transplantation, modality change, spontaneous kidney recovery, and loss to follow-up; (*2*) receiving HDF with predilution mode in which replacement fluid is infused before the dialyzer, leading to lower effective convective clearance^14^; (*3*) receiving hemodialysis or HDF <75% of treatments during the first year of follow-up; (*4*) residing in countries delivering one type of dialysis modality; and/or (*5*) having average online clearance monitoring (OCM) Kt/V value <1.2 during the baseline period (Supplemental Figure 1).

The study was conducted in accordance with the principles of the Declaration of Helsinki. Ethical approval was obtained from the Ethics Committee of the Landesärztekammer Hessen (Medical Association of Hesse) in Frankfurt, Germany. Written consent was obtained from all participants for the secondary use of their data for scientific research purposes.

### Exposure Measures

Dialysis modality data were extracted from individual treatment records in the EuCliD database. Treatments were classified as hemodialysis if recorded as “haemodialysis double needle” (*i.e*., conventional hemodialysis, including treatments delivered either by two-needle vascular access [arteriovenous fistula or graft] or by a double-lumen catheter) and as HDF if recorded as “online haemodiafiltration.” All treatments used ultrapure dialysate. HF-HD treatments were conducted using contemporary high-flux synthetic polysulfone membranes. HDF treatments were administered in postdilution mode using online-generated substitution fluid. Patients were classified into the HDF or hemodialysis group, respectively, if they received ≥75% sessions as HDF or hemodialysis during the first year of dialysis.

### Outcome Assessment

For each patient, a 30-day baseline period was defined, commencing on the date of the first recorded treatment at a Fresenius NephroCare center (*i.e*., index date), to capture baseline clinical characteristics and allow stabilization of treatment parameters. Follow-up started on day 31 and continued until the occurrence of one of the following events: death, kidney transplantation, a modality switch to peritoneal dialysis or home hemodialysis, spontaneous renal recovery, loss to follow-up, or the end of the study period. To evaluate the survival effect of HDF in patients new to dialysis, follow-up was truncated at a maximum of 2 years. The primary outcome was all-cause mortality, and the secondary outcome was cardiovascular mortality. The underlying cause of death was documented by the treating physician in the EuCliD database using International Classification of Diseases, Tenth Revision codes, on the basis of the best available information from hospitals, families, and postmortem reports, and was available for 93.4% of all deaths. Cardiovascular death was identified on the basis of relevant International Classification of Diseases, Tenth Revision codes (Supplemental Table 1).

### Statistical Analyses

Descriptive statistics were generated separately for the hemodialysis and HDF groups to summarize demographic characteristics, renal etiology, and comorbidities as of the index date. Dialysis vintage was defined as the duration from the initiation of kidney replacement therapy to the end of the baseline period. Vascular access type was determined on the basis of the predominant access used during the baseline period, defined as the access type used in ≥75% of sessions. Predialysis BP, blood flow rate, effective treatment time, and laboratory values were calculated as the mean values over the baseline period.

Inverse probability of treatment weighting (IPTW) was used to balance patient baseline characteristics between the HDF and hemodialysis groups. Propensity scores, defined as the probability of receiving HDF versus hemodialysis, were estimated using a logistic regression model. The model included covariates such as demographic characteristics, kidney failure etiology, comorbidities, dialysis vintage, vascular access type, OCM Kt/V, systolic BP, blood flow rate, and treatment time. IPTW weights were then calculated as the inverse of the propensity score for patients in the HDF group and the inverse of one minus the propensity score for patients in the hemodialysis group. Covariate balance between the HDF and hemodialysis groups was evaluated using standardized differences, assessed both before and after weighting. The average treatment effect of HDF relative to hemodialysis on all-cause mortality was estimated using IPTW-weighted Cox regression models. To eliminate the influence of extreme weights on variance of the effect estimate, stabilized IPTW was applied by incorporating the marginal probability of treatment (*e.g*., proportion of patients in the HDF group multiplied by the individual IPTW) in the numerator of the weight formula. Sensitivity analyses were conducted by (*1*) combing IPTW with covariate adjustment, including age and vascular access type, which may bear residual imbalance after IPTW; (*2*) excluding patients with extreme propensity scores (≤0.01 or ≥0.99); (*3*) alternatively defining vascular access type according to the predominant access used during follow-up; (*4*) fitting the regression model with a random effect for dialysis clinics to account for potential clinic-level heterogeneity in modality use. In an additional sensitivity analysis approximating an intention-to-treat approach, patients were classified according to dialysis modality at the end of the baseline period, irrespective of subsequent modality changes during follow-up. Competing-risk analyses were conducted using Fine–Gray subdistribution hazard models, with kidney transplantation treated as a competing event.

Subgroup analyses were performed by stratifying patients according to age (18–50, 50–65 years, and over 65 years), sex, diabetes status on the index date, history of CVD, vascular access type (fistula, catheter).

Cause-specific Cox models were applied for the secondary outcome, CVD mortality, with deaths from other causes treated as censoring events. Sensitivity analyses were performed with Fine–Gray models, treating deaths from other causes as competing events. Adjusted survival curves of the HDF and hemodialysis groups for both all-cause and CVD mortality were plotted. The proportional hazards assumption was assessed using Schoenfeld residuals and diagnostic plots, and no violations were detected for the models.

All analyses were conducted using SAS software, version 9.4 (SAS Institute, Cary, NC).

## Results

### Patient Population

**Figure 1:**
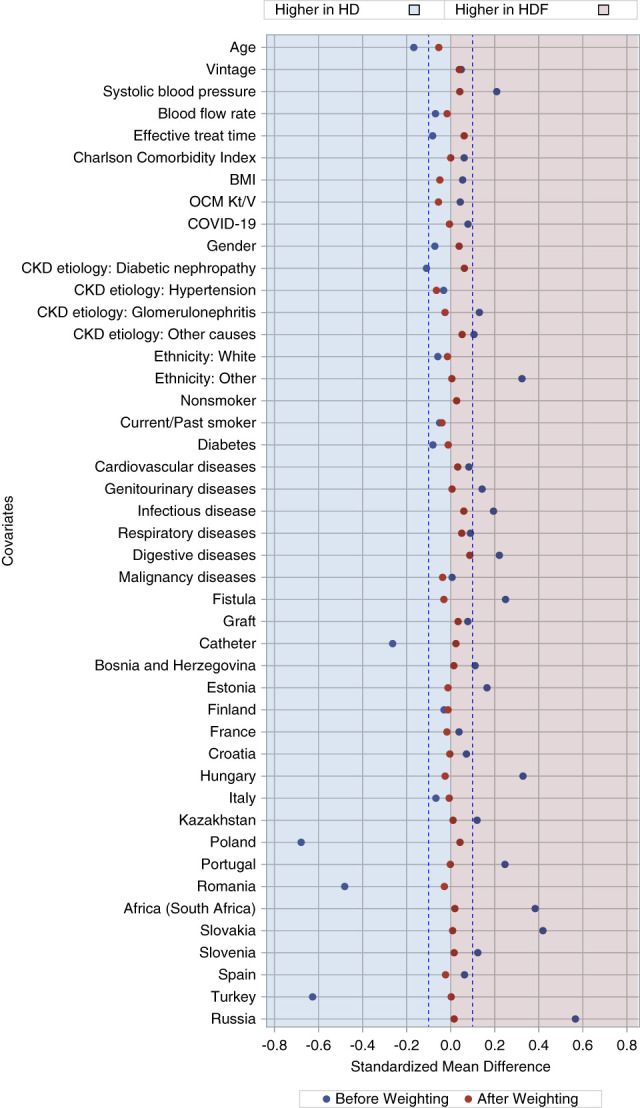
**Standardized mean differences of covariates before and after propensity score weighting.** BMI, body mass index; CCI, Charlson Comorbidity Index; COVID-19, coronavirus disease 2019; HD, hemodialysis; HDF, hemodiafiltration; OCM, online clearance monitoring; PRD, primary renal diagnosis.

A total of 18,515 patients who met the study criteria were included in the analysis (Supplemental Figure 1). Within the first year of follow-up, 10,149 (55%) patients predominantly received hemodialysis and 8366 (45%) patients received HDF (Table 1). Over a median follow-up of 15.7 months (interquartile range [IQR], 6.4–24.0 months), patients on HDF received a total of 1,507,757 postdilution HDF treatments, with a mean convection volume of 24.9 L (median [IQR], 25.1 [23.1–27.2]). HDF treatment started on average on day 7 of follow-up, *i.e*., after the baseline period (median [IQR], 1 [1–2] days). At baseline, the HDF group had a lower mean age than the hemodialysis group (62.0 [SD, 15] versus 64.5 [SD, 15] years), a higher proportion of men (58% versus 54%), a higher proportion of patients with fistula access (40% versus 28%), and a lower proportion of patients with catheter access (59% versus 72%). Charlson Comorbidity Index was similar between the two groups, while the HDF group had a lower proportion of patients with preexisting diabetes (34% versus 38%) but a higher proportion of patients with preexisting CVD (72% versus 68%). Other baseline characteristics were comparable between the hemodialysis and HDF groups, including dialysis vintage, median effective treatment time, median blood flow rate, and OCM Kt/V. After applying IPTW, all baseline characteristics were successfully balanced between the HDF and hemodialysis groups as summarized in Table 1 and with standardized mean differences <|0.10| as displayed in Figure 1.

**Table 1:** Patients' characteristics at baseline (*N*=18,515)

Variables	Before Weighting		After Weighting	
	Hemodialysis (*N*=10,149)	HDF (*N*=8366)	Hemodialysis (*N*=10,149)	HDF (*N*=8366)
Age, yr, mean±SD	64.5±14.5	62.0±15.1	63.3±15.0	62.9±15.2
Male, *n* (%)	5518 (54)	4849 (58)	5549 (55)	4599 (54)
**Ethnicity, *n* (%)**				
White	5900 (58)	4627 (55)	5574 (56)	4718 (556)
Other	209 (2)	799 (10)	487 (5)	431 (5)
Unknown	4040 (40)	2940 (35)	3939 (40)	3430 (40)
**Smoking status, *n* (%)**				
Nonsmoker	4361 (43)	3707 (44)	4159 (42)	3682 (43)
Current/past smoker	2129 (21)	1598 (19)	1984 (20)	1566 (18)
Unknown	3659 (36)	3061 (37)	3858 (39)	3331 (39)
**Kidney etiology, *n* (%)**				
Diabetes mellitus	1648 (16)	1035 (12)	1532 (15)	1520 (18)
Hypertension	1012 (10)	757 (10)	934 (9)	647 (8)
GN	870 (9)	1055 (13)	1007 (10)	798 (9)
Other causes	1361 (13)	1444 (17)	1486 (15)	1433 (17)
Unknown	5258 (52)	4075 (49)	5041 (50)	4181 (49)
CCI, mean±SD	3.7±1.8	3.8±2.0	3.8±1.9	3.8±2.0
Preexisting diabetes, *n* (%)	3828 (38)	2832 (34)	3687 (37)	3113 (36)
Preexisting CVDs, *n* (%)	6880 (68)	5993 (72)	6959 (70)	6098 (71)
**Vascular access, *n* (%)**				
Fistula	2824 (28)	3307 (40)	3149 (31)	2579 (30)
Graft	42 (0.4)	91 (1)	64 (1)	81 (1)
Catheter	7274 (72)	4951 (59)	6787 (68)	5918 (69)
Vintage, d, mean±SD	43.3±18.5	44.2±20.6	43.5±19.4	44.4±21.2
BMI, kg/m^2^, mean±SD	26.2±5.7	26.5±5.7	26.4±6.2	26.1±5.6
Systolic BP, mm Hg, mean±SD	142.6±18.2	146.5±18.6	144.4±18.4	145.2±19.6
Dialysis frequency, d/wk, mean±SD	2.9±0.3	3.0±0.3	2.9±0.3	2.9±0.3
Duration of session, min, median (IQR)	240.0 (223.7–242.4)	237.0 (219.4–242.5)	237.5 (219.4–242.1)	239.4 (223.4–242.4)
Blood flow, ml/min, median (IQR)	309.2 (273.1–337.0)	300.6 (268.4–331.8)	305.2 (269.0–336.2)	302.0 (269.9–333.8)
Heart rate before dialysis, beats/min, mean±SD	75.3±9.6	74.8±10.8	75.2±10.0	75.2±10.6
OCM Kt/V	1.4±0.3	1.4±0.3	1.4 (1.2–1.6)	1.4 (1.2–1.6)
**IDWG, kg**				
*N* miss (%)	21 (0.2)	66 (0.8)	21 (0.2)	66 (0.8)
Mean±SD	1.3±0.8	1.3±0.8	1.3±0.8	1.3±0.8

BMI, body mass index; CCI, Charlson Comorbidity Index; CVD, cardiovascular disease; HDF, hemodiafiltration; IDWG, interdialytic weight gain; IQR, interquartile range; OCM, online clearance monitoring.

### Primary Outcome

The overall mortality rate among the patients on HDF was lower than those on hemodialysis (11.7 versus 15.6 deaths per 100 person-years, Table 2). After controlling for confounding factors by IPTW, HDF was associated with a 20% lower risk of death compared with hemodialysis (hazard ratio [HR], 0.80; 95% confidence interval [CI], 0.75 to 0.86). Additional adjustment for covariates yielded a similar treatment effect (HR, 0.82; 95% CI, 0.77 to 0.88). After adjustment for dialysis clinic effects, a further lower mortality risk was observed (HR, 0.63; 95% CI, 0.57 to 0.69). In sensitivity analysis excluding patients with extreme IPTWs, HDF remained associated with a lower mortality risk (HR, 0.74; 95% CI, 0.68 to 0.79; Supplemental Table 2). Alternatively defining vascular access on the basis of the predominant type used during follow-up, a similar association with mortality risk reduction was yielded (HR, 0.78; 95% CI, 0.72 to 0.84; Supplemental Table 3). Identical association with lower mortality risk among patients treated with hemodialysis was also obtained in competing-risk analyses with transplantation as the competing event (Supplemental Table 4). Results were also consistent in the intention-to-treat–like sensitivity analysis, with HDF associated with lower mortality (HR, 0.88, 95% CI, 0.82 to 0.95).

**Table 2:** Association of hemodiafiltration relative to hemodialysis with all-cause morality

Estimates	Hemodialysis	HDF
No. of patients	10,149	8366
No. of deaths	1863	1246
Person-years	11,925	10,638
Incidence rate (100 person-years)	15.6	11.7
**Mortality risk, HR (95% CI)**		
Crude model	Reference	0.75 (0.70 to 0.81)
Stabilized IPTW^a^	Reference	0.80 (0.75 to 0.86)
Stabilized IPTW^a^+age+vascular access	Reference	0.82 (0.77 to 0.88)
Stabilized IPTW^a^+age+vascular access+IDWG	Reference	0.82 (0.77 to 0.88)
Stabilized IPTW^a^+dialysis clinic (random effect)	Reference	0.63 (0.57 to 0.69)

CI, confidence interval; HDF, hemodiafiltration; HR, hazard ratio; IDWG, interdialytic weight gain; IPTW, inverse probability of treatment weighting.

a Covariates included in inverse probability of treatment weighting are country, age, sex, ethnicity, tobacco use, renal etiology, comorbidities (including diabetes, cardiovascular disease, infectious disease, respiratory disease, digestive disease, genitourinary disease, and malignant disease), Charlson Comorbidity Index, coronavirus disease 2019, dialysis vintage, body mass index, vascular access, systolic BP, blood flow rate, effective treatment time, and online clearance monitoring Kt/V at baseline.

**Figure 2:**
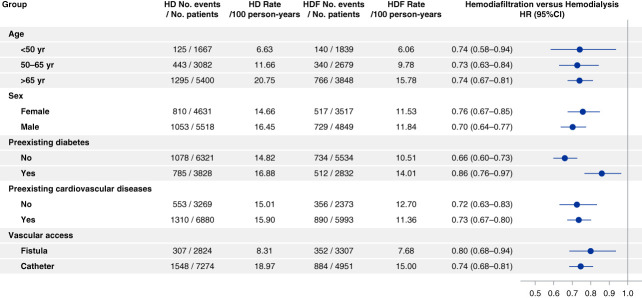
**Association of HDF relative to hemodialysis with all-cause mortality in subgroups.** HRs were estimated by a Cox regression model with IPTW; covariates included in IPTW are country, age, sex, ethnicity, tobacco use, renal etiology, comorbidities (including diabetes, CVD, infectious disease, respiratory disease, digestive disease, genitourinary disease, and malignant disease), CCI, COVID-19, dialysis vintage, BMI, vascular access, systolic BP, blood flow rate, effective treatment time, and OCM Kt/V at baseline. CI, confidence interval; CVD, cardiovascular disease; HR, hazard ratio; IPTW, inverse probability of treatment weighting.

The association between HDF and lower mortality was consistent across all subgroups analyzed (Figure 2). No statistically significant interactions between dialysis modality and subgroup variables were observed, except for diabetes status (*P* < 0.001). A stronger association with lower mortality risk was observed among incident patients without documented diabetes at baseline (HR, 0.66; 95% CI, 0.60 to 0.73).

### Secondary Outcomes

**Figure 3:**
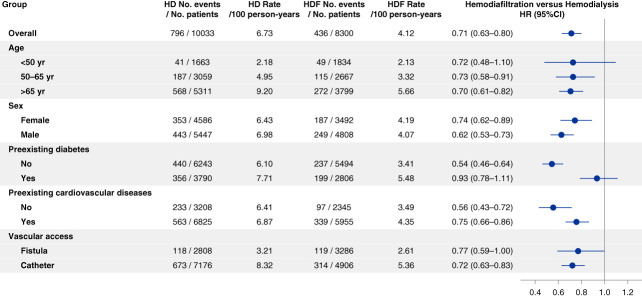
**Association of HDF relative to hemodialysis with CVD mortality.** HRs were estimated by a Cox regression model with IPTW; covariates included in IPTW are country, age, sex, ethnicity, tobacco use, renal etiology, comorbidities (including diabetes, CVD, infectious disease, respiratory disease, digestive disease, genitourinary disease, and malignant disease), CCI, COVID-19, dialysis vintage, BMI, vascular access, systolic BP, blood flow rate, effective treatment time, and OCM Kt/V at baseline.

The CVD mortality rate was 4.1 and 6.7 deaths per 100 person-years, respectively, for the HDF and hemodialysis groups (Figure 3). Overall, HDF was associated with lower CVD mortality compared with hemodialysis (HR, 0.71; 95% CI, 0.63 to 0.80). This association was also consistent across all subgroups, except among patients with comorbid diabetes at baseline, where statistical significance was not reached. Similar results were obtained in competing-risk analysis (Supplemental Table 5).

### Survival Curves

**Figure 4:**
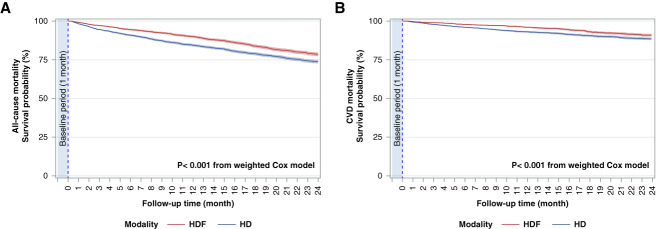
**Adjusted survival curves by dialysis modality.** (A) All-cause mortality. (B) CVD mortality.

For both primary and secondary outcomes, adjusted survival curves of the HDF and hemodialysis groups began to diverge shortly after the start of follow-up, with the HDF group consistently demonstrating higher survival probabilities compared with the hemodialysis group throughout the study period (Figure 4).

## Discussion

In this large, international, multicenter, retrospective cohort study using IPTW, we demonstrated that HDF was associated with 20% lower all-cause mortality and 29% lower CVD mortality, compared with conventional hemodialysis. Notably, the separation of adjusted survival curves emerged shortly after the initiation of dialysis and persisted throughout the follow-up period. These associations were consistent across multiple demographic and clinical subgroups. These findings accord well with data derived from prior clinical trials in prevalent dialysis populations^3,6–8^ and extend the evidence base to patients at the onset of dialysis therapy.

Evidence for a survival advantage of HDF over HF-HD has primarily been derived from randomized clinical trials conducted in predominantly prevalent patients who have been on dialysis for a longer duration.^3,5–8^ For example, although eligibility criteria varied across studies, the median dialysis vintage in the HDF arm was 35 months in the CONVINCE trial and 27 months in Estudio de Supervivencia de Hemodiafiltración OnLine trial,^5,6^ and the mean vintage in FRENCH Convective versus Hemodialysis In the Elderly and the Turkish trial were approximately 5 years.^7,8^ By contrast, data in patients newly starting dialysis are limited to a small number of observational studies.^15–18^ Maduell *et al.* propensity score matched 1012 patients on dialysis for less than 3 months and observed lower hazards with HDF versus hemodialysis for all-cause mortality (HR, 0.76; 95% CI, 0.62 to 0.94) and CVD mortality (HR, 0.67; 95% CI, 0.50 to 0.90).^18^ In another propensity score‑matched study, Canaud *et al.* reported 50% lower mortality with HV-HDF (≥21 L substitution volume) compared with hemodialysis.^16^ A French nationwide incident cohort identified 33% lower mortality among 2254 patients treated exclusively with HDF.^17^ Consistent patterns from these studies and our large multicenter study suggest that applying HDF among incident patients may also confer a survival benefit. Notably, all prior studies were based on incident patients with follow-up time up to 4–8 years; therefore, the estimated effects of HDF reflected a mixture of short- and long-term treatment. In comparison, our study targeted the early phase of dialysis by limiting follow-up time up to maximum of 2 years. In addition, 75% of patients on HDF in this study had their first HDF treatment before or in 1 to 2 days after the start of follow-up, and we observed survival curves diverged shortly thereafter. Our findings extend the existing evidence and additionally support early-stage adoption of HDF.

HDF offers superior clearance of both small solutes and larger middle molecules by combining diffusion with convection, which allows effective removal of proinflammatory and proatherogenic molecules, such as *β*2-microglobulin, cytokines, and advanced glycation end products.^19–21^ Mechanistically, this enhanced solute clearance has important implications for the cardiovascular system, such as lowering systemic inflammation and improving endothelial health.^22,23^ Although the CONVINCE trial did not demonstrate a statistically significant reduction in CVD mortality, this trial enrolled a selected prevalent population with a relatively lower baseline cardiovascular risk profile (*e.g*., <22% coronary artery disease and <47% any CVD). Notably, pooled analyses of randomized clinical trials, including CONVINCE, have demonstrated substantially lower risk of CVD mortality with HDF among prevalent patients.^3,6^ Focusing on the relatively early stage of incident patients in the current study, we observed a similar magnitude of association with lower CVD mortality, which bears a particular clinical significance because these patients are in a period marked by highly elevated cardiovascular morbidity and mortality. Our findings may suggest that initiating HDF among incident patients potentially has a positive influence on the CVD-related disease trajectory and therefore has the potential to decrease the disease burden at the individual patient and health care system levels.

This study has several notable strengths. The large, multicenter, international sample represented a diverse patient population treated in real-world practice. Methodology rigor was enhanced through using IPTW to control for confounding, complemented by sensitivity analyses that consistently supported the robustness of results. A further strength lies in the focus on incident patients and the evaluation of HDF treatment effect in the early phase of dialysis. However, several limitations should be acknowledged. First, our analysis was restricted to incident patients with sufficient follow-up and meeting guideline-recommended dialysis adequacy criteria. As a result, patients experiencing early censoring events or inadequate dialysis delivery were excluded, which may limit generalizability to more clinically stable incident dialysis populations and may not fully extend to patients with early treatment instability or very high short-term risk. In addition, compared with the patients on HD in the United States,^24–26^ patients in this study had a lower prevalence of diabetes, lower arteriovenous fistula use, lower interdialytic weight gain, lower blood flow rates, and a longer treatment session. These differences should be considered when extrapolating our findings to US hemodialysis populations. Furthermore, as an observational study based on real-world data, residual confounding from unmeasured variables cannot be excluded despite careful adjustments. The earlier separation of survival curves observed in this incident cohort compared with randomized trials conducted in prevalent populations may reflect not only biologic differences related to early dialysis initiation but also residual confounding. In addition, some degree of nondifferential misclassification inherent to routinely collected electronic health care records is possible, including for cause-of-death assignment. Finally, the observational design precludes definitive causal inference, emphasizing the need for randomized trials to confirm these findings specifically in incident patients on dialysis.

In conclusion, our study demonstrates that initiating HDF shortly after starting dialysis is associated with lower all-cause and cardiovascular mortality across diverse patient subgroups. These findings suggest early adoption of HDF may have clinical benefit when vascular access and residual kidney function are relatively preserved. Future prospective RCTs focusing on incident patients on dialysis are warranted to verify these survival advantages and clarify mechanisms. Ultimately, such evidence could inform clinical guidelines and translate into meaningful survival gains at a stage when patients on dialysis are most vulnerable.

## Supplementary Material

## Data Availability

Original data generated for the study will be made available upon reasonable request to the corresponding author. Data Type: Health Care Data. Reason for Restricted Access: The datasets analyzed in the current study are not publicly available due to confidential protected patient information. Further inquiries can be addressed with the corresponding author.

## Supplemental Material

Supplemental Figure 1. Study flow chart.

Supplemental Table 1. International Classification of Diseases, 10th Revision, codes for CVD.

Supplemental Table 2. Association of HDF relative to hemodialysis with all-cause mortality after trimming patients with 0.01< propensity score <0.99.

Supplemental Table 3. Association of HDF relative to hemodialysis with all-cause mortality in sensitivity analysis with adjustment for predominant type of vascular access during follow-up.

Supplemental Table 4. Association of HDF relative to hemodialysis with all-cause mortality in competing-risk analysis.

Supplemental Table 5. Association of HDF relative to hemodialysis with CVD mortality in competing-risk analysis.

## References

[1] Thurlow JS Joshi M Yan G, . Global epidemiology of end-stage kidney disease and disparities in kidney replacement therapy. Am J Nephrol. 2021;52(2):98–107. doi:10.1159/000514550 33752206 PMC8057343

[2] Wolley MJ Hutchison CA. Large uremic toxins: an unsolved problem in end-stage kidney disease. Nephrol Dial Transplant. 2018;33(suppl 3):iii6–11. doi:10.1093/ndt/gfy179 30281131 PMC6168891

[3] Vernooij RWM Hockham C Strippoli G, . Haemodiafiltration versus haemodialysis for kidney failure: an individual patient data meta-analysis of randomised controlled trials. Lancet. 2024;404(10464):1742–1749. doi:10.1016/s0140-6736(24)01859-2 39489903

[4] Stuard S Maddux FW. High-volume hemodiafiltration: expanding the evidence beyond randomized trials-a critical perspective on the 2025 EuDial consensus. J Clin Med. 2025;14(9):3174. doi:10.3390/jcm14093174 40364203 PMC12072281

[5] Blankestijn PJ Vernooij RWM Hockham C, .; CONVINCE Scientific Committee Investigators. Effect of hemodiafiltration or hemodialysis on mortality in kidney failure. N Engl J Med. 2023;389(8):700–709. doi:10.1056/NEJMoa2304820 37326323

[6] Maduell F Moreso F Pons M, . High-efficiency postdilution online hemodiafiltration reduces all-cause mortality in hemodialysis patients. J Am Soc Nephrol. 2013;24(3):487–497. doi:10.1681/ASN.2012080875 23411788 PMC3582206

[7] Ok E Asci G Toz H, . Mortality and cardiovascular events in online haemodiafiltration (OL-HDF) compared with high-flux dialysis: results from the Turkish OL-HDF study. Nephrol Dial Transplant. 2013;28(1):192–202. doi:10.1093/ndt/gfs407 23229932

[8] Morena M Jaussent A Chalabi L, . Treatment tolerance and patient-reported outcomes favor online hemodiafiltration compared to high-flux hemodialysis in the elderly. Kidney Int. 2017;91(6):1495–1509. doi:10.1016/j.kint.2017.01.013 28318624

[9] Chan KE Maddux FW Tolkoff-Rubin N Karumanchi SA Thadhani R Hakim RM. Early outcomes among those initiating chronic dialysis in the United States. Clin J Am Soc Nephrol. 2011;6(11):2642–2649. doi:10.2215/CJN.03680411 21959599 PMC3359565

[10] Karaboyun K Girgin S Yilmaz M. 90 day and 1-year mortality and renal outcomes of patients who started hemodialysis treatment for the first time. Rev Nefrol Dial Transplant. 2023;43(2):69–78.

[11] Heaf J Heiro M Petersons A, . First-year mortality in incident dialysis patients: results of the peridialysis study. BMC Nephrol. 2022;23(1):229. doi:10.1186/s12882-022-02852-1 35761193 PMC9235232

[12] Marcelli D Kirchgessner J Amato C, . EuCliD (European Clinical Database): a database comparing different realities. J Nephrol. 2001;14(suppl 4):S94–S100. PMID: 1179815 11798154

[13] Barbieri C Neri L Stuard S Mari F Martin-Guerrero JD. From electronic health records to clinical management systems: how the digital transformation can support healthcare services. Clin Kidney J. 2023;16(11):1878–1884. doi:10.1093/ckj/sfad168 37915897 PMC10616428

[14] Lang T Zawada AM Theis L, . Hemodiafiltration: technical and medical insights. Bioengineering (Basel). 2023;10(2):145. doi:10.3390/bioengineering10020145 36829639 PMC9952158

[15] Imamovic G Hrvacevic R Kapun S, . Survival of incident patients on high-volume online hemodiafiltration compared to low-volume online hemodiafiltration and high-flux hemodialysis. Int Urol Nephrol. 2014;46(6):1191–1200. doi:10.1007/s11255-013-0526-8 24057682

[16] Canaud B Bayh I Marcelli D, . Improved survival of incident patients with high-volume haemodiafiltration: a propensity-matched cohort study with inverse probability of censoring weighting. Nephron. 2015;129(3):179–188. doi:10.1159/000371446 25765538

[17] Mercadal L Franck JE Metzger M, . Hemodiafiltration versus hemodialysis and survival in patients with ESRD: the French renal epidemiology and information network (REIN) registry. Am J Kidney Dis. 2016;68(2):247–255. doi:10.1053/j.ajkd.2015.11.016 26724836

[18] Maduell F Varas J Ramos R, . Hemodiafiltration reduces all-cause and cardiovascular mortality in incident hemodialysis patients: a propensity-matched cohort study. Am J Nephrol. 2017;46(4):288–297. doi:10.1159/000481669 29041011

[19] Pellicano R Polkinghorne KR Kerr PG. Reduction in beta2-microglobulin with super-flux versus high-flux dialysis membranes: results of a 6-week, randomized, double-blind, crossover trial. Am J Kidney Dis. 2008;52(1):93–101. doi:10.1053/j.ajkd.2008.02.296 18423807

[20] Roumelioti ME Trietley G Nolin TD, . Beta-2 microglobulin clearance in high-flux dialysis and convective dialysis modalities: a meta-analysis of published studies. Nephrol Dial Transplant. 2018;33(6):1025–1039. doi:10.1093/ndt/gfx311 29186592

[21] Blankestijn PJ Ledebo I Canaud B. Hemodiafiltration: clinical evidence and remaining questions. Kidney Int. 2010;77(7):581–587. doi:10.1038/ki.2009.541 20130529

[22] Stuard S Maddux FW Canaud B. Why high-volume post-dilution hemodiafiltration should be the new standard in dialysis care: a comprehensive review of clinical outcomes and mechanisms. J Clin Med. 2025;14(14):4860. doi:10.3390/jcm14144860 40725552 PMC12295283

[23] Canaud B Blankestijn PJ Grooteman MPC Davenport A. Why and how high volume hemodiafiltration may reduce cardiovascular mortality in stage 5 chronic kidney disease dialysis patients? A comprehensive literature review on mechanisms involved. Semin Dial. 2022;35(2):117–128. doi:10.1111/sdi.13039 34842306

[24] Kerr PG. International differences in hemodialysis delivery and their influence on outcomes. Am J Kidney Dis. 2011;58(3):461–470. doi:10.1053/j.ajkd.2011.04.021 21783291

[25] Pisoni RL Zepel L Zhao J, . International comparisons of native arteriovenous fistula patency and time to becoming catheter-free: findings from the dialysis outcomes and practice patterns study (DOPPS). Am J Kidney Dis. 2021;77(2):245–254. doi:10.1053/j.ajkd.2020.06.020 32971192

[26] Saran R Bragg-Gresham JL Levin NW, . Longer treatment time and slower ultrafiltration in hemodialysis: associations with reduced mortality in the DOPPS. Kidney Int. 2006;69(7):1222–1228. doi:10.1038/sj.ki.5000186 16609686

