# Supplementary material for: Hemodialysis Modality and Mortality Outcomes among Incident Dialysis Patients: An International Cohort Study Comparing High-Volume Hemodiafiltration and Hemodialysis
Source: Clin J Am Soc Nephrol. 2026 May 14;21(7):1198–206. doi: 10.2215/CJN.0000001063 (PMC13379127; doi:10.2215/CJN.0000001063)
Supplement: Supplementary file 1 [file cjasn-21-1198-s001.pdf]

ASN American Society of Nephrology
JASN Journal of the American Society of Nephrology
KIDNEY360 Accessing Our World From Every Angle
CJASN Clinical Journal of the American Society of Nephrology

ASN Journal Disclosure Form

As per ASN journal policy, I have disclosed any financial relationships or commitments I have held in the past 36 months as included below. I have listed my Current Employer below to indicate there is a relationship requiring disclosure. If no relationship exists, my Current Employer is not listed.

S. Arya reports the following:
Employer: Fresenius Medical Care

I understand that the information above will be published within the journal article, if accepted, and that failure to comply and/or to accurately and completely report the potential financial conflicts of interest could lead to the following: 1) Prior to publication, article rejection, or 2) Post-publication, sanctions ranging from, but not limited to, issuing a correction, reporting the inaccurate information to the authors' institution, banning authors from submitting work to ASN journals for varying lengths of time, and/or retraction of the published work.

Name: Smriti Arya
Manuscript ID: CJASN-2025-001922
Manuscript Title: Hemodialysis modality and all-cause mortality among incident dialysis patients: An international cohort study comparing high-volume hemodiafiltration and hemodialysis
Date of Completion: January 29, 2026
Disclosure Updated Date: January 29, 2026

ASN
American Society of Nephrology
JASN
Journal of the American Society of Nephrology
KIDNEY360
Accessing Our World From Every Angle
CJASN
Clinical Journal of the American Society of Nephrology

ASN Journal Disclosure Form

As per ASN journal policy, I have disclosed any financial relationships or commitments I have held in the past 36 months as included below. I have listed my Current Employer below to indicate there is a relationship requiring disclosure. If no relationship exists, my Current Employer is not listed.

L. Ficociello reports the following:
Employer: Renal Research Institute, which is a wholly owned subsidiary of Fresenius Medical Care; and Ownership Interest: Fresenius Medical Care.

I understand that the information above will be published within the journal article, if accepted, and that failure to comply and/or to accurately and completely report the potential financial conflicts of interest could lead to the following: 1) Prior to publication, article rejection, or 2) Post-publication, sanctions ranging from, but not limited to, issuing a correction, reporting the inaccurate information to the authors' institution, banning authors from submitting work to ASN journals for varying lengths of time, and/or retraction of the published work.

Name: Linda Ficociello
Manuscript ID: CJASN-2025-001922
Manuscript Title: Hemodialysis modality and all-cause mortality among incident dialysis patients: An international cohort study comparing high-volume hemodiafiltration and hemodialysis
Date of Completion: February 5, 2026
Disclosure Updated Date: October 15, 2025

ASN American Society of Nephrology | JASN Journal of the American Society of Nephrology | KIDNEY360 Accessing Our World From Every Angle | CJASN Clinical Journal of the American Society of Nephrology

ASN Journal Disclosure Form

As per ASN journal policy, I have disclosed any financial relationships or commitments I have held in the past 36 months as included below. I have listed my Current Employer below to indicate there is a relationship requiring disclosure. If no relationship exists, my Current Employer is not listed.

K. Kalantar-Zadeh reports the following:
Employer: 1) Los Angeles County Department of Health Services (LAC-DHS) including LAC Harbor-UCLA, 2) Veterans Administration (VA), 3) The Lundquist Institute at Harbor-UCLA; Consultancy: Kabi (2026), Abbott (2026); Research Funding: 1) NIH NIDDK, 2) VA ORD, 3) Lundquist Institute at Harbor-UCLA; Honoraria: 1) Agency for Healthcare Research and Quality (AHRQ) including study section membership, 2) National Institutes of Health (NH), 3) Kabi, 4) Abbott; Patents or Royalties: PATENTS:; 1. Kalantar-Zadeh K, Caulfield MP and Salameh WA. Prognostic assays for maintenance hemodialysis patients. 2019 (Harbor-UCLA/Lundquist); 2. Moradi H, Piomelli D and Kalantar-Zadeh K. Methods of treating renal disease. 2021 (Univ. Calif. Irvine, UCI); and Advisory or Leadership Role: 1. National Forum of the ESRD Networks, President (2023-2025) & Board of Directors (since 2017), coalition of 18 US congressionally mandated ESRD networks; 2. Expert Panel/co-chair: Batelle's PQM (Partnership for Quality Measurements) CMS Measure Set Review. 3. Co-Chair, CMS TEP for Bones and Minerals, ESRD QIP, 2024-2025.

I understand that the information above will be published within the journal article, if accepted, and that failure to comply and/or to accurately and completely report the potential financial conflicts of interest could lead to the following: 1) Prior to publication, article rejection, or 2) Post-publication, sanctions ranging from, but not limited to, issuing a correction, reporting the inaccurate information to the authors' institution, banning authors from submitting work to ASN journals for varying lengths of time, and/or retraction of the published work.

Name: Kamyar Kalantar-Zadeh
Manuscript ID: CJASN-2025-001922
Manuscript Title: Hemodialysis modality and mortality outcomes among incident dialysis patients: An international cohort study comparing high-volume hemodiafiltration and hemodialysis
Date of Completion: April 30, 2026
Disclosure Updated Date: April 30, 2026

ASN
American Society of Nephrology
JASN
Journal of the American Society of Nephrology
KIDNEY360
Accessing Our World From Every Angle
CJASN
Clinical Journal of the American Society of Nephrology

ASN Journal Disclosure Form

As per ASN journal policy, I have disclosed any financial relationships or commitments I have held in the past 36 months as included below. I have listed my Current Employer below to indicate there is a relationship requiring disclosure. If no relationship exists, my Current Employer is not listed.

S. Stuard reports the following:
Employer: Fresenius Medical Care; Ownership Interest: Fresenius Medical Care; and Patents or Royalties: Fresenius Medical Care.

I understand that the information above will be published within the journal article, if accepted, and that failure to comply and/or to accurately and completely report the potential financial conflicts of interest could lead to the following: 1) Prior to publication, article rejection, or 2) Post-publication, sanctions ranging from, but not limited to, issuing a correction, reporting the inaccurate information to the authors' institution, banning authors from submitting work to ASN journals for varying lengths of time, and/or retraction of the published work.

Name: Stefano Stuard
Manuscript ID: CJASN-2025-001922R1
Manuscript Title: Hemodialysis modality and mortality outcomes among incident dialysis patients: An international cohort study comparing high-volume hemodiafiltration and hemodialysis
Date of Completion: January 30, 2026
Disclosure Updated Date: October 11, 2025

ASN American Society of Nephrology | JASN Journal of the American Society of Nephrology | KIDNEY360 Accessing Our World From Every Angle | CJASN Clinical Journal of the American Society of Nephrology

ASN Journal Disclosure Form

As per ASN journal policy, I have disclosed any financial relationships or commitments I have held in the past 36 months as included below. I have listed my Current Employer below to indicate there is a relationship requiring disclosure. If no relationship exists, my Current Employer is not listed.

L. Usvyat reports the following:
Employer: Renal Research Institute, LLC; Ownership Interest: Fresenius Medical Care; Research Funding: Investigator on several grants awarded to Renal Research Institute, including R01DK130067, R01HS029890-01A1, and others.; and Advisory or Leadership Role: Executive Advisory Board Member for Privacy Analytics Inc, a Member of the AWS Research Customer Advisory Council, a Steering Committee Member on the MONitoring Dialysis Outcomes (MONDO) initiative, and a Steering Committee Member on the American Society of Nephrology Partnership for Responsible Augmented Intelligence in Kidney Health. Invited Working Group Participant for the KDIGO Controversies Conference.

I understand that the information above will be published within the journal article, if accepted, and that failure to comply and/or to accurately and completely report the potential financial conflicts of interest could lead to the following: 1) Prior to publication, article rejection, or 2) Post-publication, sanctions ranging from, but not limited to, issuing a correction, reporting the inaccurate information to the authors' institution, banning authors from submitting work to ASN journals for varying lengths of time, and/or retraction of the published work.

Name: Len A. Usvyat
Manuscript ID: CJASN-2025-001922
Manuscript Title: Hemodialysis modality and all-cause mortality among incident dialysis patients: An international cohort study comparing high-volume hemodiafiltration and hemodialysis
Date of Completion: January 29, 2026
Disclosure Updated Date: January 29, 2026

ASN American Society of Nephrology | JASN Journal of the American Society of Nephrology | KIDNEY360 Accessing Our World From Every Angle | CJASN Clinical Journal of the American Society of Nephrology

ASN Journal Disclosure Form

As per ASN journal policy, I have disclosed any financial relationships or commitments I have held in the past 36 months as included below. I have listed my Current Employer below to indicate there is a relationship requiring disclosure. If no relationship exists, my Current Employer is not listed.

A. Winter reports the following:
Employer: Fresenius Medical Care Deutschland GmbH

I understand that the information above will be published within the journal article, if accepted, and that failure to comply and/or to accurately and completely report the potential financial conflicts of interest could lead to the following: 1) Prior to publication, article rejection, or 2) Post-publication, sanctions ranging from, but not limited to, issuing a correction, reporting the inaccurate information to the authors' institution, banning authors from submitting work to ASN journals for varying lengths of time, and/or retraction of the published work.

Name: Anke Winter
Manuscript ID: CJASN-2025-001922
Manuscript Title: Hemodialysis modality and all-cause mortality among incident dialysis patients: An international cohort study comparing high-volume hemodiafiltration and hemodialysis
Date of Completion: January 30, 2026
Disclosure Updated Date: January 30, 2026

ASN American Society of Nephrology | JASN Journal of the American Society of Nephrology | KIDNEY360 Accessing Our World From Every Angle | CJASN Clinical Journal of the American Society of Nephrology

ASN Journal Disclosure Form

As per ASN journal policy, I have disclosed any financial relationships or commitments I have held in the past 36 months as included below. I have listed my Current Employer below to indicate there is a relationship requiring disclosure. If no relationship exists, my Current Employer is not listed.

Y. Zhang reports the following:
Employer: Fresenius Medical Care

I understand that the information above will be published within the journal article, if accepted, and that failure to comply and/or to accurately and completely report the potential financial conflicts of interest could lead to the following: 1) Prior to publication, article rejection, or 2) Post-publication, sanctions ranging from, but not limited to, issuing a correction, reporting the inaccurate information to the authors' institution, banning authors from submitting work to ASN journals for varying lengths of time, and/or retraction of the published work.

Name: Yan Zhang
Manuscript ID: CJASN-2025-001922
Manuscript Title: Hemodialysis modality and all-cause mortality among incident dialysis patients: An international cohort study comparing high-volume hemodiafiltration and hemodialysis
Date of Completion: January 29, 2026
Disclosure Updated Date: January 29, 2026
