# Supplementary material for: Hemodialysis Modality and Mortality Outcomes among Incident Dialysis Patients: An International Cohort Study Comparing High-Volume Hemodiafiltration and Hemodialysis
Source: Clin J Am Soc Nephrol. 2026 May 14;21(7):1198–206. doi: 10.2215/CJN.0000001063 (PMC13379127; doi:10.2215/CJN.0000001063)
Supplement: Supplementary file 2 [file cjasn-21-1198-s002.pdf]

SUPPLEMENTAL MATERIAL

Table of contents

Supplementary Figure 1. Study flowchart

Supplementary Table 1. International Classification of Diseases, 10th Revision codes for cardiovascular disease

Supplementary Table 2. Association of HDF relative to HD with all-cause mortality after trimming patients with 0.01< propensity score<0.99

Supplementary Table 3. Association of HDF relative to HD with all-cause mortality in sensitivity analysis with adjustment for predominant type of vascular access during follow-up

Supplementary Table 4. Association of HDF relative to HD with all-cause mortality in competing risk analysis

Supplementary Table 5. Association of HDF relative to HD with CVD mortality in competing risk analysis

Supplementary Figure 1. Study flowchart

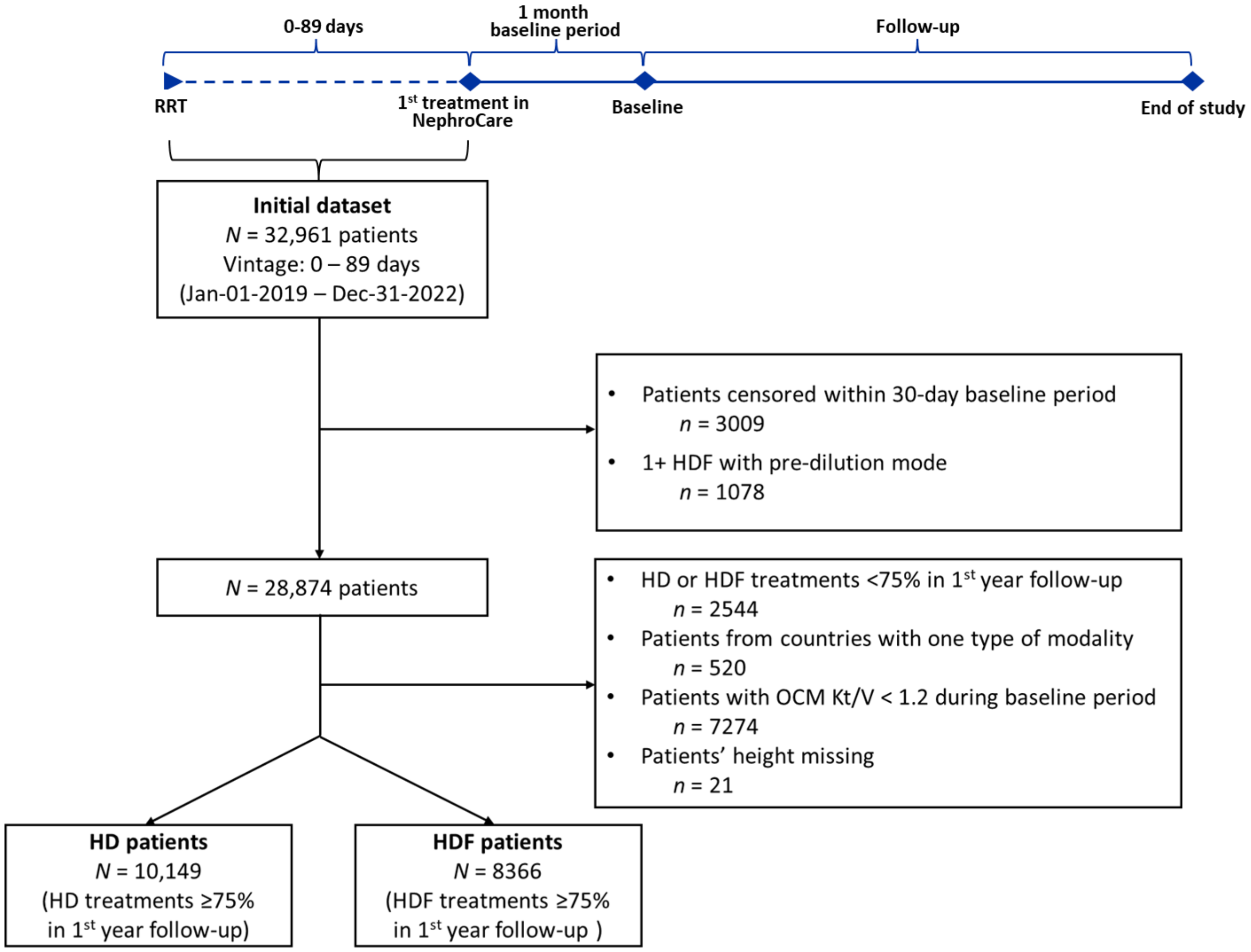

Supplementary Table 1. International Classification of Diseases, 10th Revision codes for cardiovascular disease

**Cardiovascular Disease Codes**
E10.5, E11.5, E12.5, E13.5, E14.5, G45, G45.0, G45.1, G45.2, G45.3, G45.4, G45.8, G45.9, G46, G46.0, G46.1, G46.2, G46.3, G46.4, G46.5, G46.6, G46.7, G46.8, H34, H34.0, H34.1, H34.2, H34.8, H34.9, I00, I01, I01.0, I01.1, I01.2, I01.8, I01.9, I02.0, I05, I05.0, I05.1, I05.2, I05.8, I05.9, I06, I06.0, I06.1, I06.2, I06.8, I06.9, I07, I07.0, I07.1, I07.2, I07.8, I07.9, I08, I08.0, I08.1, I08.2, I08.3, I08.8, I08.9, I09, I09.0, I09.1, I09.2, I09.8, I09.9, I10, I11, I11.0, I11.9, I12, I12.0, I12.9, I13, I13.0, I13.1, I13.2, I13.9, I15, I15.0, I15.1, I15.2, I15.8, I15.9, I20, I20.0, I20.1, I20.8, I20.9, I21, I21.0, I21.1, I21.2, I21.3, I21.4, I21.9, I22, I22.0, I22.1, I22.8, I22.9, I23, I23.0, I23.1, I23.2, I23.3, I23.4, I23.5, I23.6, I23.8, I24, I24.0, I24.1, I24.8, I24.9, I25, I25.0, I25.1, I25.2, I25.3, I25.4, I25.5, I25.6, I25.8, I25.9, I26, I26.0, I26.9, I27, I27.0, I27.1, I27.2, I27.8, I27.9, I28, I28.0, I28.1, I28.8, I28.9, I30, I30.0, I30.1, I30.8, I30.9, I31, I31.0, I31.1, I31.2, I31.3, I31.8, I31.9, I32, I32.0, I32.1, I32.8, I33, I33.0, I33.9, I34, I34.0, I34.1, I34.2, I34.8, I34.9, I35, I35.0, I35.1, I35.2, I35.8, I35.9, I36, I36.0, I36.1, I36.2, I36.8, I36.9, I37, I37.0, I37.1, I37.2, I37.8, I37.9, I38, I39, I39.0, I39.1, I39.2, I39.3, I39.4, I39.8, I40, I40.0, I40.1, I40.8, I40.9, I41, I41.0, I41.1, I41.2, I41.8, I42, I42.0, I42.1, I42.2, I42.3, I42.4, I42.5, I42.6, I42.7, I42.8, I42.9, I43, I43.0, I43.1, I43.2, I43.8, I44, I44.0, I44.1, I44.2, I44.3, I44.4, I44.5, I44.6, I44.7, I45, I45.0, I45.1, I45.2, I45.3, I45.4, I45.5, I45.6, I45.8, I45.9, I46, I46.0, I46.1, I46.9, I47, I47.0, I47.1, I47.2, I47.9, I48, I48.0, I48.1, I48.2, I48.3, I48.4, I48.9, I49, I49.0, I49.1, I49.2, I49.3, I49.4, I49.5, I49.8, I49.9, I50, I50.0, I50.1, I50.9, I51, I51.0, I51.1, I51.2, I51.3, I51.4, I51.5, I51.6, I51.7, I51.8, I51.9, I52, I52.0, I52.1, I52.8, I60, I60.0, I60.1, I60.2, I60.3, I60.4, I60.5, I60.6, I60.7, I60.8, I60.9, I61, I61.0, I61.1, I61.2, I61.3, I61.4, I61.5, I61.6, I61.8, I61.9, I62, I62.0, I62.1, I62.9, I63, I63.0, I63.1, I63.2, I63.3, I63.4, I63.5, I63.6, I63.8, I63.9, I64, I65, I65.0, I65.1, I65.2, I65.3, I65.8, I65.9, I66, I66.0, I66.1, I66.2, I66.3, I66.4, I66.8, I66.9, I67, I67.0, I67.1, I67.2, I67.3, I67.4, I67.5, I67.6, I67.7, I67.8, I67.9, I68, I68.0, I68.1, I68.2, I68.8, I69, I69.0, I69.1, I69.2, I69.3, I69.4, I69.8, I70, I70.0, I70.1, I70.2, I70.8, I70.9, I71, I71.0, I71.1, I71.2, I71.3, I71.4, I71.5, I71.6, I71.8, I71.9, I72, I72.0, I72.1, I72.2, I72.3, I72.4, I72.5, I72.6, I72.8, I72.9, I73, I73.0, I73.1, I73.8, I73.9, I74, I74.0, I74.1, I74.2, I74.3, I74.4, I74.5, I74.8, I74.9, I77, I77.0, I77.1, I77.2, I77.3, I77.4, I77.5, I77.6, I77.8, I77.9, I78, I78.0, I78.1, I78.8, I78.9, I79, I79.0, I79.1, I79.2, I79.8, I80, I80.0, I80.1, I80.2, I80.3, I80.8, I80.9, I81, I82, I82.0, I82.1, I82.2, I82.3, I82.8, I82.9, I83, I83.0, I83.1, I83.2, I83.9, I85, I85.0, I85.9, I86, I86.0, I86.1, I86.2, I86.3, I86.4, I86.8, I87, I87.0, I87.1, I87.2, I87.8, I87.9, I88, I88.0, I88.1, I88.8, I88.9, I89, I89.0, I89.1, I89.8, I89.9, I95, I95.0, I95.1, I95.2, I95.8, I95.9, I97, I97.0, I97.1, I97.2, I97.8, I97.9, I98, I98.0, I98.1, I98.2, I98.3, I98.8, I99, K76.1, K76.2, M31.8, M31.9, O22.5, O22.8, O22.9, O87.3, O87.8, O87.9, O90.3, O99.4, P29.0, R00.0, R00.1, R00.2, T81.7

Supplementary Table 2. Association of HDF relative to HD with all-cause mortality after trimming patients with 0.01< propensity score<0.99

Model	HDF versus HD<br>HR [95% CI]
Crude model	0.77 (0.72−0.83)
Stabilized IPTW[*]	0.74 (0.68−0.79)
Stabilized IPTW[*] + age + vascular access	0.76 (0.70−0.82)
Stabilized IPTW[*] + age + vascular access + IDWG	0.76 (0.70−0.82)

[*]Covariates included in IPTW are country, age, gender, ethnicity, tobacco use, renal etiology, comorbidities (including diabetes, cardiovascular disease, infectious disease, respiratory disease, digestive disease, genitourinary disease, malignant disease), Charlson Comorbidity Index, COVID-19, dialysis vintage, body mass index, vascular access, systolic blood pressure, blood flow rate, effective treatment time, OCM Kt/V at baseline.

CI, confidence interval; HD, hemodialysis; HDF, hemodiafiltration; IPTW, inverse probability of treatment weighting; HR, hazard ratio.

Supplementary Table 3. Association of HDF relative to HD with all-cause mortality in sensitivity analysis with adjustment for predominant type of vascular access during follow-up

Model	HDF versus HD<br>HR [95% CI]
Stabilized IPTW*	0.78 (0.72−0.84)
Stabilized IPTW* + age + vascular access	0.79 (0.73−0.85)
Stabilized IPTW* + age + vascular access + IDWG	0.80 (0.74−0.86)

*Covariates included in IPTW are country, age, gender, ethnicity, tobacco use, renal etiology, comorbidities (including diabetes, cardiovascular disease, infectious disease, respiratory disease, digestive disease, genitourinary disease, malignant disease), Charlson Comorbidity Index, COVID-19, dialysis vintage, body mass index, systolic blood pressure, blood flow rate, effective treatment time, OCM Kt/V at baseline, and predominant type of vascular access during follow-up.

CI, confidence interval; HD, hemodialysis; HDF, hemodiafiltration; IPTW, inverse probability of treatment weighting; HR, hazard ratio.

Supplementary Table 4. Association of HDF relative to HD with all-cause mortality in competing risk analysis

Model	HDF versus HD<br>HR [95% CI]
Crude model	0.75 (0.70−0.81)
Stabilized IPTW*	0.80 (0.75−0.86)
Stabilized IPTW* + age + vascular access	0.82 (0.77−0.88)
Stabilized IPTW* + age + vascular access + IDWG	0.82 (0.77−0.88)

*Covariates included in IPTW are country, age, gender, ethnicity, tobacco use, renal etiology, comorbidities (including diabetes, cardiovascular disease, infectious disease, respiratory disease, digestive disease, genitourinary disease, malignant disease), Charlson Comorbidity Index, COVID-19, dialysis vintage, body mass index, vascular access, systolic blood pressure, blood flow rate, effective treatment time, OCM Kt/V at baseline.

CI, confidence interval; HD, hemodialysis; HDF, hemodiafiltration; IPTW, inverse probability of treatment weighting; HR, hazard ratio.

Supplementary Table 5. Association of HDF relative to HD with CVD mortality in competing risk analysis

Model	HDF versus HD HR [95% CI]
Overall	0.72 (0.64−0.82)
Age	
<50 yr	0.74 (0.49−1.12)
50–65 yr	0.74 (0.59−0.93)
>65yr	0.72 (0.62−0.84)
Sex	
Female	0.76 (0.63−0.91)
Male	0.64 (0.55−0.75)
Preexisting diabetes	
No	0.56 (0.47−0.66)
Yes	0.95 (0.80−1.13)
Preexisting cardiovascular disease	
No	0.57 (0.44−0.73)
Yes	0.78 (0.68−0.89)
Vascular access	
Fistula	0.78 (0.60−1.02)
Catheter	0.74 (0.64−0.85)

*Covariates included in IPTW are country, age, gender, ethnicity, tobacco use, renal etiology, comorbidities (including diabetes, cardiovascular disease, infectious disease, respiratory disease, digestive disease, genitourinary disease, malignant disease), Charlson Comorbidity Index, COVID-19, dialysis vintage, body mass index, vascular access, systolic blood pressure, blood flow rate, effective treatment time, OCM Kt/V at baseline.

CI, confidence interval; HD, hemodialysis; HDF, hemodiafiltration; IPTW, inverse probability of treatment weighting; HR, hazard ratio.
